# Contribution to the genus *Balcha* Walker (Hymenoptera, Chalcidoidea, Eupelmidae) of China, with description of a new species and DNA data

**DOI:** 10.3897/zookeys.1289.199128

**Published:** 2026-08-13

**Authors:** Qiyan Wang, Haoran Liao, Zixuan Li, Yuntao Zhang, Lingfei Peng

**Affiliations:** 1 Biological Control Research Institute, Fujian Agriculture and Forestry University, Fuzhou 350002, China South China Botanical Garden, Chinese Academy of Sciences Guangzhou China; 2 China Fruit Fly Research and Control Center of FAO/IAEA, Fuzhou 350002, China Biological Control Research Institute, Fujian Agriculture and Forestry University Fuzhou China; 3 State Key Laboratory of Agricultural and Forestry Biosecurity, Fuzhou 350002, China China Fruit Fly Research and Control Center of FAO/IAEA Fuzhou China; 4 South China Botanical Garden, Chinese Academy of Sciences, Guangzhou 510650, China State Key Laboratory of Agricultural and Forestry Biosecurity Fuzhou China

**Keywords:** *
Agrilus
*, emerald ash borer, *
Fraxinus
*, parasitoid, taxonomy

## Abstract

Eight species of *Balcha* Walker (Hymenoptera, Chalcidoidea, Eupelmidae) are reported from China, including one new species, *B. fulgida* Wang & Peng, **sp. nov**., and three newly recorded species: *B. indica* (Mani & Kaul, 1973), *B. laciniosa* Gibson, 2005, and *B. reticulata* (Nikol’skaya, 1952). An identification key to the eight Chinese species and the mitochondrial cytochrome c oxidase subunit I (COI) gene sequences for *B. dictyota* Gibson, 2005, *B. indica*, and the new species are provided. Furthermore, morphological, molecular, and host distribution lines of evidence indicate that the Nearctic population of *B. indica*, a parasitoid of the emerald ash borer, *Agrilus planipennis* Fairmaire, 1888 (Coleoptera, Buprestidae), most likely originated from China rather than from Southeast Asia as previously hypothesized. In addition, extensive intraspecific morphological variation within *B. dictyota* are documented and new host associations reported for *B. indica* and *B. reticulata*.

## Introduction

*Balcha* Walker, 1862 is native to the Old World except for one species, *B. indica* (Mani & Kaul, 1973) that was accidentally introduced to North America (Gibson 1997). Gibson (2005) revised the world species of *Balcha* primarily to identify the North American species parasitizing the emerald ash borer, *Agrilus planipennis* Fairmaire, 1888 (Coleoptera, Buprestidae), which he determined to be *B. indica* and speculated that it originated from Vietnam or elsewhere in Southeast Asia. Gibson (2005) also subdivided the 16 species recognized at that time into four species groups based on dorsellar structure, and the setal patterns of the posterior surface of dorsellum and paraspiracular region. Subsequently, Fusu et al. (2018a, 2018b) described a new species, *B. opaca* Fusu, 2018, from South Korea and assigned it to the laciniosa species group. They also reported *B. dictyota* Gibson, 2005 from South Korea for the first time based on two specimens (one female and one male), and these specimens exhibit conspicuous intraspecific variation compared to the single female holotype from Beijing.

Prior to this study, four species of *Balcha* had been recorded from China: *B. dictyota*, *B. elegans* (Masi, 1927), *B. enoptra* Gibson, 2005 and *B. eximia* (Masi, 1927) (Masi 1927; Gibson 2005). In this study, four additional species are reported from China, including one new species and three new records. This raises the total number of *Balcha* species known from China to eight, establishing China as the region with the highest documented diversity of this genus globally. To facilitate accurate identification of *Balcha* species in China, we provide an identification key, which is revised from Gibson’s (2005) framework, supplemented by the material examined in this study, and incorporates the description of *B. dictyota* by Fusu et al. (2018a, 2018b). Additionally, we have deposited the mitochondrial cytochrome c oxidase subunit I (COI) gene sequences of *B. dictyota*, *B. fulgida* Wang & Peng, sp. nov., and *B. indica* in GenBank. Finally, we discuss the origin of the Nearctic population of *B. indica*, the observed intraspecific variation of *B. dictyota*, and provide additional biological data for *B. indica* and *B. reticulata* (Nikol’skaya, 1952).

## Materials and methods

### Specimens

A total of twelve specimens were examined in this study. Specimens were collected in 2015–2025 using three methods: Malaise trapping, direct field collection, and laboratory rearing. Specimens intended for DNA extraction were preserved in 95% ethanol and stored at –20 °C prior to molecular extractions. Specimens processed for DNA extraction at Fujian Agriculture and Forestry University were assigned unique DNA voucher numbers in the format “FAFU-XXXX”. DNA extraction for the single specimen of *B. fulgida* was conducted at the South China Botanical Garden, with its corresponding voucher ID designated as SCBG-E0015534. Two specimens of *B. reticulata* from Benxi, Liaoning Province (voucher IDs: BX-1, BX-2) and three specimens of *B. dictyota* from Chuzhou, Anhui Province (voucher IDs: CZ-1, CZ-2, CZ-3) were not processed for DNA extraction, and were directly prepared as pin-mounted dry specimens. Additionally, field photographs of *B. dictyota* and *B. indica* by Xinyu Wang were also examined. All specimens are deposited in the Biological Control Research Institute, Fujian Agriculture and Forestry University, Fuzhou, Fujian, China (**FAFU**). New distribution records presented in this paper are indicated by an asterisk (*).

### Imaging

Descriptions were based on specimens examined with a Leica M165C stereo microscope (Leica Microsystems AG, Heerbrugg, Switzerland) illuminated with a Leica LED 5000 HDI domelight source and imaged with a Leica MC170 HD digital camera attached to the microscope. The serial images obtained were combined with Zerene Stacker 1.04 software (Zerene Systems, LLC, Richland, Washington, DC, USA). However, the images of specimens designated as “CZ-1, -2” were produced using a Nikon SMZ25 microscope with a Nikon DS-Ri 2 digital camera system (Nikon Corp., Tokyo, Japan). Adobe Photoshop CC2019 and Adobe Lightroom Classic (Adobe Systems Incorporated, Los Angeles, CA, USA) were used to edit pictures and enhance their clarity.

Coloration of specimens may differ between figures and direct observation due to variation in imaging equipment. All color descriptions in the text are based on direct stereomicroscopic observation, which represents the actual specimen features more accurately. Figures serve as a visual reference only.

### Morphological terms and abbreviations

Structural terms follow Fusu et al. (2018a) except supraclypeal area is used as defined in Burks et al. (2025), the style of the descriptions follow Gibson (2005), and surface sculpture terms follows Burks et al. (2025). The following abbreviations are used in the description:

**Fl** Flagellar segment;

**Gt** Gastral tergum.

### Mapping distribution

To visualize the geographical distribution of *Balcha* species across China, a distribution map was generated using QGIS (version 3.38.3), an open-source Geographic Information System software. All geographic coordinates were converted to the WGS 84 coordinate system to maintain uniformity. The base map was obtained from Tianditu (www.tianditu.gov.cn), the official national online map service of China, which provides high-resolution satellite imagery and geographic data. The Tianditu map layer was integrated into QGIS using the XY Tiles function, facilitating a comprehensive visualization of the species’ distribution. The distribution data were derived from the specimens examined in this study and the records reported by Gibson (2005).

### DNA extraction, amplification, and sequencing

DNA extraction was performed using a DNeasy Blood & Tissue Kit (Qiagen, Hilden, Germany) following the manufacturer’s instructions, with three modifications: (i) incubation was prolonged to at least 12 h at 56 °C in a thermo-shaker; (ii) the lids of the adsorption columns were left open and incubated at room temperature for 3–5 min. This modification was implemented to completely air-dry the residual ethanol inside the columns, thereby preventing any adverse impacts on DNA solubilization and the quality of subsequent PCR amplification; (iii) given the relatively low DNA content of samples, after transferring the adsorption column to a new centrifuge tube, 40 μL of Buffer AE was added dropwise to the center of the adsorption membrane without touching it, followed by incubation at room temperature for 5 min to ensure complete DNA dissolution. The mixture was then centrifuged at 12,000 rpm for 2 min. The COI gene fragment was amplified using the primers LCO1490 (5'-GGTCAACAAATCATAAAGATATTGG-3') and HCO2198 (5'-TAAACTTCAGGGTGACCAAAAAATCA-3') (Folmer et al. 1994).

PCR amplifications were conducted in a 25-µL reaction volume containing 12.5 µL of 2×SuperTaq PCR StarMix (Dye) (Beijing Kangrun Chengye Biotechnology, Beijing, China), 8.5 µL of sterile water (Beijing Kangrun Chengye Biotechnology, Beijing, China), 1 µL of each primer (10 µM each), and 2 µL of DNA template. The PCR conditions were as follows: initial denaturation at 95 °C for 2 min, followed by 35 cycles of 95 °C for 15 s, 50 °C for 15 s, and 72 °C for 15 s, with a final extension at 72 °C for 5 min. PCR products were submitted directly to Tsingke Biotech (Beijing, China) for sequencing.

### Sequence data analyses

Sequence quality verification, assembly, and editing were performed in Geneious Prime® 2021.1.1, with all COI gene sequences translated into amino acids to confirm no premature stop codons. Valid sequences were aligned using the MUSCLE algorithm with default parameters in MEGA7 (Kumar et al. 2016), and pairwise Kimura 2-parameter (K2P) genetic distances were calculated for intra- and interspecific comparisons (Kimura 1980). Reference COI gene sequences of *Balcha indica* from other geographic regions were obtained from the Barcode of Life Data Systems (BOLD Systems; Table 1), and all new valid COI gene sequences generated in this study were deposited in GenBank (Table 1).

**Table 1.** COI gene sequences of *Balcha* species with voucher information and GenBank accession numbers.

**Taxon**	**Voucher ID**	**Locality**	**GenBank accession no**.
*Balcha dictyota* Gibson	FAFU-1073	China: Jiangsu	PZ022429
*Balcha dictyota* Gibson	FAFU-1645	China: Jiangxi	PZ022432
*Balcha fulgida* Wang & Peng, sp. nov.	SCBG-E0015534	China: Guangdong	PZ022431
*Balcha indica* (Mani & Kaul)	FAFU-1428	China: Jiangsu	PZ022430
*Balcha indica* (Mani & Kaul)	CNCHYM-011879	Canada: Ontario	—
*Balcha indica* (Mani & Kaul)	CNCHYM-011880	USA: Maryland	—
*Balcha indica* (Mani & Kaul)	CNCHYM-011882	USA: Kentucky	—
*Balcha indica* (Mani & Kaul)	CNCHYM-011883	USA: Minnesota	—

Notes: All sequences were obtained from female specimens. Sequences with FAFU or SCBG prefixes were newly generated in this study and deposited in GenBank. Sequences with CNCHYM prefixes are BOLD Sample IDs downloaded from BOLD Systems and have no corresponding GenBank accession numbers.

## Results

### Sequence analysis

The COI gene sequences of three *Balcha* species were successfully amplified and sequenced. After alignment with the COI gene sequences of *B. indica* from different geographical regions downloaded from BOLD Systems, the pairwise K2P interspecific genetic distances among the three species ranged from 9.4–14.5% (Table 2). Intraspecific K2P genetic distances of the species examined herein were 0–0.2% for *B. indica* and 6.5% for *B. dictyota*.

**Table 2.** Pairwise K2P genetic distances of COI gene sequences of *Balcha* species.

**Taxa & Voucher ID**	1	2	3	4	5	6	7	8
1. dic FAFU-1645	0							
2. dic FAFU-1073	0.065	0						
3. ind FAFU-1428	0.135	0.128	0					
4. ind CNCHYM-011879	0.145	0.117	0.002	0				
5. ind CNCHYM-011880	0.145	0.117	0.002	0.000	0			
6. ind CNCHYM-011882	0.145	0.117	0.002	0.000	0.000	0		
7. ind CNCHYM-011883	0.145	0.117	0.002	0.000	0.000	0.000	0	
8. ful SCBG-E0015534	0.094	0.098	0.132	0.128	0.128	0.128	0.128	0

Notes: dic = *Balcha dictyota* Gibson, 2005; ind = *Balcha indica* (Mani & Kaul, 1973); ful = *Balcha fulgida* Wang & Peng, sp. nov.

### Taxonomic account

#### Balcha

Taxon classificationAnimaliaHymenopteraEupelmidae

Walker, 1862

702CA0F2-A71C-5472-A34F-9F5150E71E72

Balcha Walker, 1862: 394. Type species Balcha cylindrica Walker, by monotypy. Type locality The Natural History Museum, London, UK.
Elemba Cameron, 1908: 151. Type species Elemba levicollis Cameron, by monotypy. Type locality The Natural History Museum, London, UK. Synonymy by Hedqvist (1961: 109).
Sauteria Masi, 1927: 333–334. Type species Sauteria eximia Masi, by original designation. Type locality Deutsches Entomologisches Institut, Eberswalde, Germany. Synonymy by Bouček (1988: 544).

##### Diagnosis.

Macropterous. Antenna 13-segmented; clava 3-segmented. Mesoscutum relatively broad, wider than long; in anterior half with submedian, linear, subparallel notaular and similar anteroadmedian and parapsidal lines paralaterally; usually patterned with finely sculptured and darker colored regions relative to more grossly sculptured and brilliantly metallic regions. Scutellum quadrangular to rectangular; axilla elongate triangular to almost linear along extreme anterolateral corner of scutellum. Prepectus distinctly separated from base of tegula. Acropleuron conspicuously alveolate anteriorly and more finely though variably sculptured posteriorly. Gaster with Gt_7_ and Gt_8_ fused into syntergum.

##### Distribution.

Native: Afrotropical (Democratic Republic of the Congo, Kenya, South Africa, Tanzania, Zambia), Australasian region (Indonesia, Papua New Guinea), Oriental (Brunei, China, India, Indonesia, Malaysia, Myanmar, Philippines, Singapore, Sri Lanka, Thailand, Vietnam), and Palaearctic (China, Russia, South Korea). Introduced: Nearctic (Canada, USA).

##### Species groups.

See Gibson (2005).

### Key to Chinese species of *Balcha* Walker

**Table:** 

1	Propodeum with paraspiracular region setose (Figs 1C, 2F)	**2 (elegans species group)**
–	Propodeum with paraspiracular region bare (Figs 3D, 4D, 5D)	**5**
2	Face with punctures brightly colored in contrast to dark interstices (Fig. 3B); petiole without longitudinal crenulation (Fig. 3D)	***B. elegans* (Masi, 1927)**
–	Face with punctures and interstices of similar color, except sometimes parascrobal region with darker black to coppery regions (Figs 1B, 2B); petiole with longitudinal crenulation (Figs 1C, 2F)	**3**
3	Parascrobal region with bright blue to purple metallic luster, without darker region; lower face with distinct punctures, even near oral margin; scutellum varying from coriaceous to punctate-coriaceous or with sculpture arranged into more or less distinct longitudinal setiferous furrows	***B. eximia* (Masi, 1927)**
–	Parascrobal region multicolored with contrasting darker regions (Figs 1B, 2B, 2C); lower face punctate-reticulate near oral margin (Figs 1B, 2B); scutellum reticulate (Figs 1D, 1E, 2E)	**4**
4	Both sexes: mesoscutum dorsally patterned with dark coppery bands and brilliant green and blue metallic lusters (Fig. 1D, E); parascrobal region with two dark coppery regions (Fig. 1B); scrobal depression distinctly sculptured ventrally (Fig. 1B)	***B. dictyota* Gibson, 2005**
–	Female only known: mesoscutum entirely with brilliant reddish and golden metallic lusters (Fig. 2E); parascrobal region with one dark coppery region (Fig. 2B); scrobal depression almost smooth ventrally (Fig. 2C)	***B. fulgida* Wang & Peng, sp. nov**.
5	Dorsellum with posterior surface setose (Fig. 3D, cf. Gibson 2005: fig. 48)	...***B. indica* (Mani & Kaul, 1973) (cylindrica species group)**
–	Dorsellum with posterior surface bare (Figs 4D, 5D)	**6 (laciniosa species group)**
6	Both sexes: mesoscutum with distinct, lanceolate white setae laterally and dorsomedially within anteroadmedian band (Fig. 4C)	***B. laciniosa* Gibson, 2005**
–	Female only known: mesoscutum with slightly lanceolate white setae only laterally (Fig. 5C)	**7**
7	Syntergum with dorsal surface smooth, shiny and bare posterior to basal setose band (Gibson 2005: fig. 55); scutellum dark with violaceous luster or slight greenish tinge under some angles of light; coriaceous-granular with sculpture mostly aligned into shallow, setiferous furrows and irregular, low, rounded longitudinal ridges, and with tiny punctures near posterior margin (Gibson 2005: fig. 42)	***B. enoptra* Gibson, 2005**
–	Syntergum uniformly sculptured and setose (Figs 4E, 5E); scutellum mostly greenish with variably distinct coppery luster; reticulate (Fig. 5C)	***B. reticulata* (Nikol’skaya, 1952)**

### Descriptions

#### Balcha dictyota

Taxon classificationAnimaliaHymenopteraEupelmidae

Gibson, 2005

4EB3EFE3-8F42-59F8-9F5B-D91F603B3F13

Figs 1, 6A

Balcha dictyota Gibson, 2005: 20–22.
Balcha dictyota : Fusu et al. 2018a: 329–334; Fusu et al. 2018b: 2605–2607.

##### Material examined.

**China** • 1♀; Jiangsu Province, Nanjing City, Jiangning District, Tangshan Scenic Area; 12 May. 2024; Fan Gao leg.; FAFU-1073 • 3♀♀; Anhui Province, Chuzhou City, Nanqiao District, Chuzhou University (Huifeng Campus); 29 May. 2024; Hui Liu & Fan Zhang leg.; Malaise trap • 1♀; Jiangxi Province, Nanchang City, Meiling, Nanchang Botanical Garden; alt. ~ 300 m; 24 Oct. 2025; reared from wood; FAFU-1645.

**Figure 1.**
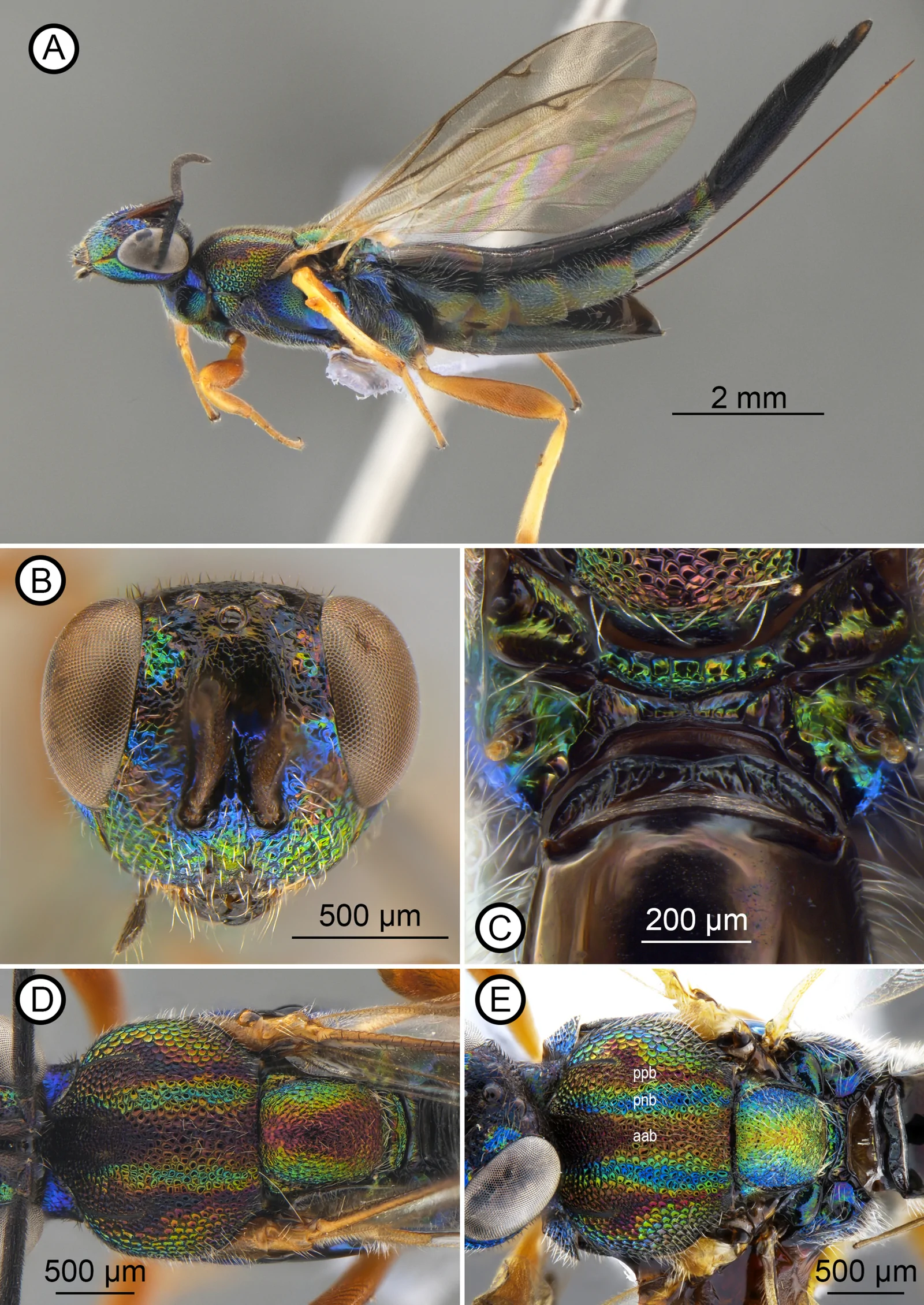
*Balcha dictyota* Gibson, 2005. **A**. Body, lateral; **B**. Head, frontal; **C**. Metanotum and propodeum; **D**. Mesosoma, dorsal; **E**. Mesosoma, dorsal (**A, D** from CZ-1; **B, C** from FAFU-1073; **E** from CZ-2) Abbreviations: aab = anteroadmedian band, pnb = paranotaular band, ppb = parapsidal band.

##### Photograph.

**China** • 1♂; Jiangsu Province, Nanjing City, 13 April. 2025, photo by Xinyu Wang.

##### Diagnosis.

**Female**. Face (Fig. 1B) with punctures and interstices the same color except parascrobal region with two dark coppery regions, lower face punctate-reticulate towards oral margin. Scrobal depression (Fig. 1B) reticulate ventrally to reticulate-coriaceous dorsally. Mesoscutum (Fig. 1D, E) with anteroadmedian and parapsidal bands dark coppery, extending posteriorly, and either separate or only indistinctly joined near anterior one-third of length; with white hairlike setae. Scutellum (Fig. 1D, E) medially varying from predominantly dark coppery to reduced, paler coppery, and progressively green to blue or purple laterally; reticulate. Dorsellum (Fig. 1C) thick with posterior surface bare. Propodeum (Fig. 1C) with paraspiracular region setose. Fore wing (Fig. 1A) hyaline. Petiole (Fig. 1C) with longitudinal crenulation.

##### Distribution.

China (*Anhui, Beijing, *Jiangsu, *Jiangxi), South Korea.

##### Biology.

Unknown.

##### Remarks.

The original description of *Balcha dictyota* was based on a single female from Beijing, characterized by longitudinal parapsidal bands extending over approximately half the mesoscutal length and a green scutellum (Gibson 2005, fig. 15). Specimens collected from southern China in this study exhibit intraspecific variation in mesothoracic coloration (Fig. 1D, E) similar to that of the female specimen reported from South Korea (Fusu et al. 2018a, 2018b), while differing from the holotype. Furthermore, obvious intraspecific variation in mesothoracic coloration is also observed among these southern Chinese specimens: one individual from Chuzhou, Anhui Province (Fig. 1E), has narrower anteroadmedian and parapsidal bands than others, such that its paranotaular bands appear conspicuously broader. Its scutellum also displays reduced, paler coppery color medially, with brighter coloration laterally. This morphology represents a clear transitional state along a continuum of intraspecific variation, bridging the morphological characters of the holotype and other examined specimens.

Additionally, the specimens from southern China differ conspicuously in body length, ranging from 7.5–16.8 mm. One large specimen from Nanchang, Jiangxi Province possesses a conspicuously more elongate and tapered syntergum. The remaining specimens reach a maximum body length of only ~ 10.4 mm, forming two largely discrete size classes rather than a continuous size gradient. This body length variation is presumably attributable to differences in host resource availability during larval development, which may be associated with different host species or host instars.

#### Balcha fulgida

Taxon classificationAnimaliaHymenopteraEupelmidae

Wang & Peng sp. nov.

A01B419C-67DA-5953-A703-D5D0B004E325

https://zoobank.org/238754F2-32CE-4EC2-92A7-9B475B0F5985

Fig. 2

##### Type material.

***Holotype*** (FAFU): **China** • ♀; Guangdong province, Zhaoqing City, Dinghu District, Dinghu Mountain National Nature Reserve masson pine forest; 23°09'39"N, 112°32'20"E; alt. 162 m; 29 Feb.–29 Mar. 2024; Qinggong Mao leg.; Malaise trap; SCBG-E0015534.

**Figure 2.**
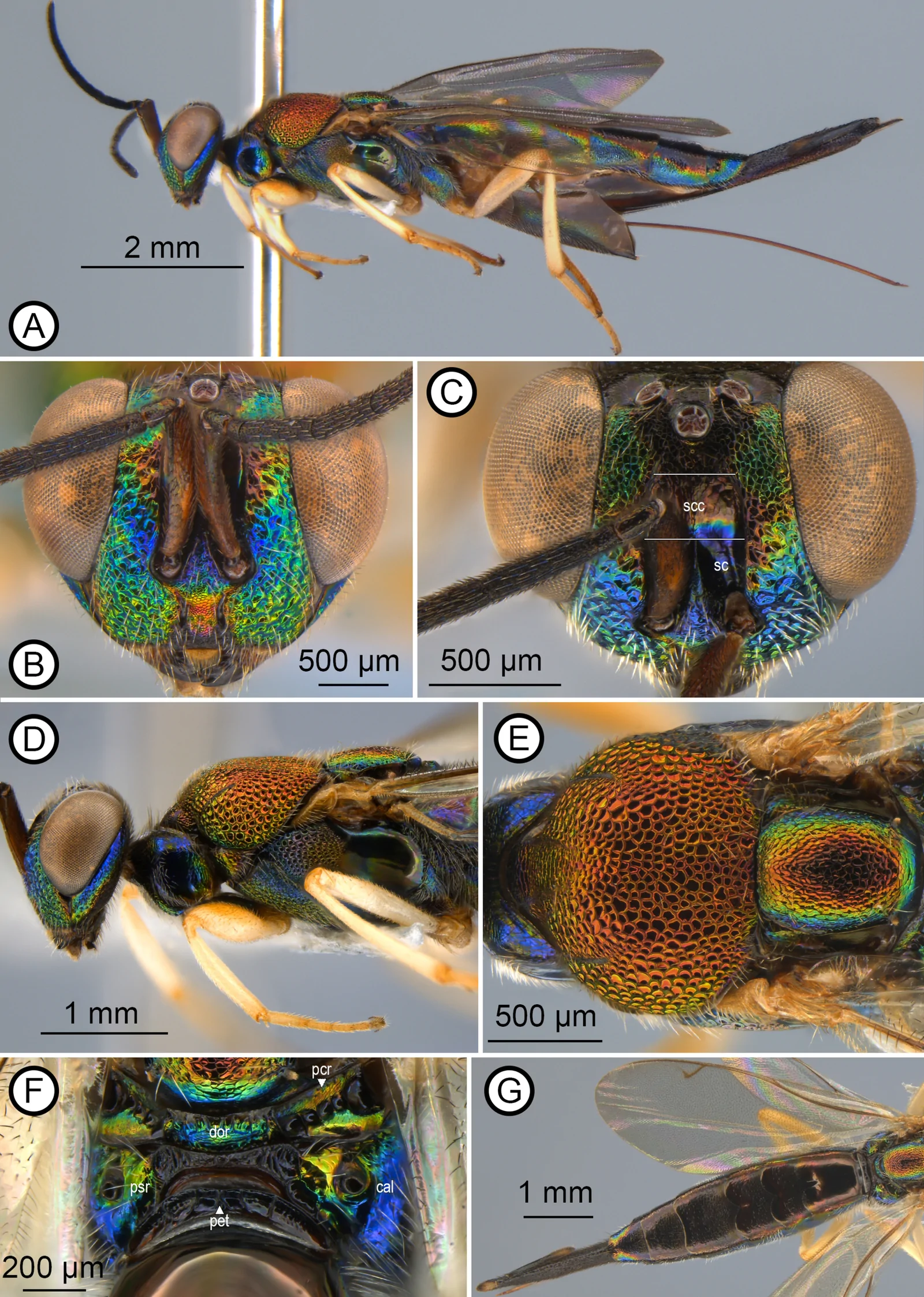
*Balcha fulgida* Wang & Peng, sp. nov. (holotype, SCBG-E0015534). **A**. Body, lateral; **B**. Head, frontal; **C**. Head, fronto-dorsal showing scrobal depression; **D**. Head and mesosoma, lateral; **E**. Mesosoma, dorsal; **F**. Metanotum and propodeum; **G**. Fore wing and gaster, dorsal. Abbreviations: cal = callus; dor = dorsellum; pcr = precrenular region of metanotal panel; pet = petiole; psr = paraspiracular region; sc = scrobe; scc = scrobal channel.

##### Diagnosis.

**Female**. Face (Fig. 2B) with punctures and interstices the same color except parascrobal region with one dark coppery region, lower face punctate-reticulate towards oral margin. Scrobal depression (Fig. 2C) almost smooth and shiny ventrally to reticulate dorsally. Mesoscutum (Fig. 2E) entirely with brilliant reddish and golden metallic luster; with slightly lanceolate white setae. Scutellum (Fig. 2E) dark medially, otherwise with brilliant metallic luster; reticulate. Dorsellum (Fig. 2F) thick with posterior surface bare. Propodeum (Fig. 2F) with paraspiracular region setose. Fore wing (Fig. 2G) hyaline. Petiole (Fig. 2F) with longitudinal crenulation.

##### Description.

**Female**. Length ~ 9 mm.

***Head***. Antenna with scape dark brown, pedicel (Fig. 2B) with green metallic luster and flagellum dark; scape uniformly setose, with apical ~1/2 slightly wider; Fl_1_ only slightly longer than apical width and ~1/2 as long as pedicel; Fl_2_ ~ 1.1 × as long as clava. Head (Fig. 2B) with punctures and interstices not contrasting in color, the face variably green to purple under different angles of light except parascrobal region with dark coppery region, dorsally between scrobal channel and inner orbit at juncture of punctate and more finely sculptured region, and supraclypeal area coppery; ocellar region and vertex dark, but occiput and outer orbit (Fig. 2D) violaceous to green under some angles of light. Face (Fig. 2B) with shallow and closely crowded punctures with mostly linear interstices so as to appear punctate-reticulate, but medially on parascrobal region with punctures separated by flat, finely coriaceous interstices, and dorsal parascrobal region reticulate; uniformly setose with white setae. Scrobal depression (Fig. 2C) with scrobes (Fig. 2C: sc) almost smooth and shiny, dark or with blue luster under some angles of light; scrobal channel (Fig. 2C: scc) ventrally blue to purple, smooth and dorsally dark coppery, reticulate.

***Mesosoma***. Pronotum (Fig. 2D, E) dark with slight reddish to green luster anteriorly and more distinctly blue, to violaceous, to green metallic luster posteriorly and laterally under some angles of light; coriaceous to alutaceous. Tegula (Fig. 2D, E) yellow. Mesoscutum (Fig. 2D, E) with golden to green metallic luster laterally; dorsally with three reddish metallic separate longitudinal bands: anteroadmedian band extending posteriorly to scutellum and parapsidal bands extending posteriorly to axilla; the bands separated by paranotaular bands with indistinct golden metallic luster; the region between reddish and golden metallic luster with orange metallic luster transition. Mesoscutum (Fig. 2E) alveolate laterally and reticulate dorsally, and much finer in region between notaular and near parapsidal line; with very shallow, obscure longitudinal depression anterior to level of inner margin of axilla; with slightly lanceolate white setae laterally and dorsally (setae mostly absent dorsomedially but the dark spots on mesoscutum likely represent pores of setae that have been lost). Scutellum (Fig. 2E) dark medially but with progressively reddish to blue metallic luster laterally and posteriorly; shallowly, reticulate; setose similar to mesoscutum. Metanotum (Fig. 2F) dark with green to blue luster under some angles of light; dorsellum (Fig. 2F: dor) thick, with crenulate dorsal surface and coriaceous, bare, posterior surface; precrenular region of metanotal panel (Fig. 2F: pcr) with single line of setae near anterior margin. Acropleuron (Fig. 2D) with minutely and very finely coriaceous, inconspicuous subalar region separating alveolate prealar region from finely alutaceous postalar region; prealar region graduating from blue-violet luster anteriorly to golden-green posteriorly, subalar and postalar regions dark with violaceous to greenish-blue luster under some angles of light. Lower mesepimeron reticulate. Metapleuron reticulate except for crenulate furrow along anterior margin ventrally, densely setose. Propodeum (Fig. 2F) with paraspiracular region (Fig. 2F: psr) golden-green, callus (Fig. 2F: cal) blue to violaceous dorsally and laterally, and plical region dark; paraspiracular region setose; callus shiny and smooth between setal pores; plical region bare, crenulate, with carinate margin of foramen ∩-like. Fore wing (Fig. 2G) hyaline; vannal area with subcubital line of setae extending over apical ~1/2. Legs (Fig. 2A) uniformly pale yellow beyond coxae.

***Metasoma***. Petiole (Fig. 2F: pet) composed of anterior carina and lunate horizontal surface obviously longer medially than propodeum, the surface longitudinally crenulate and crenulae distinctly stronger and more widely spaced than on propodeal plical region. Gaster (Fig. 2A, G) in dorsal view dark brown or with slight coppery luster, and all terga in lateral view and Gt_6_ in dorsal view with dark coppery to blue luster posterolaterally; ~ 1.75 × as long as head and mesosoma combined. Syntergum (Fig. 2A, G) measured dorsally ~ 0.4 × length of remaining gaster and in lateral view ~ 5 × as long as high; uniformly setose, sculptured and tapered posteriorly, with cercus at basal margin. Ovipositor sheath (Fig. 2G) dark except apex yellowish-brown.

**Male**. Unknown.

##### Etymology.

The species name is derived from the Latin word *fulgidus* (shining, glittering), referring to the entire mesoscutum exhibiting a brilliant metallic luster. The gender of the epithet matches the feminine genus *Balcha*.

##### Distribution.

China (Guangdong).

##### Biology.

Unknown.

##### Remarks.

*Balcha fulgida* is assigned to the elegans species group based on its thick dorsellum and setose paraspiracular region. This group also includes *B. elegans* (Masi, 1927), *B. dictyota*, *B. eximia* (Masi, 1927), and *B. eximiassita* Gibson, 2005. The female of *B. fulgida* can be distinguished from *B. elegans* by its petiole (Fig. 2F) having longitudinal crenulation and face (Fig. 2B) with punctures and interstices of similar color; from *B. dictyota* by its smooth and shiny scrobes and parascrobal region (Fig. 2C) with only one dark coppery region; from *B. eximia* and *B. eximiassita* by its reticulate scutellum (Fig. 2E) and lower face (Fig. 2B) that is punctate-reticulate towards the oral margin. Furthermore, *B. fulgida* can be readily distinguished from all other *Balcha* species by its entire mesoscutum dorsally (Fig. 2E) being brilliant metallic reddish-golden. Notably, for this distinctive mesoscutal metallic luster, the golden metallic luster of the paranotaular bands is often less distinct and may appear blended with the adjacent areas. This is likely due to the combined visual effect of the vivid reddish bands and the intervening orange transitional luster, rendering the golden bands less easily demarcated.

*B. fulgida* is described based on a single female. Nevertheless, given that *B. dictyota* and *B. indica* exhibit notable intraspecific morphological variation (see respective diagnoses and remarks for each species, as well as the Discussion), we emphasize that identification of *B. fulgida* should be based on an integrated evaluation of multiple morphological characters. Specimens should not be excluded from this species solely due to differences in one or a few characters relative to the original description. Additional material of *B. fulgida* from future collections will be necessary to comprehensively assess its full range of intraspecific variation.

### New record

#### Balcha indica

Taxon classificationAnimaliaHymenopteraEupelmidae

(Mani & Kaul, 1973)

3ADAE41F-B342-5218-925A-0D24FF95AB68

Figs 3, 6B

Thaumasura indica Mani & Kaul, 1973: 57–60.
Thaumasura indica : Bouček et al. 1979: 459.
Balcha indica : Gibson 1989: 67; Gibson 2005: 31–36; Tamm et al. 2025: 217–228.

##### Material examined.

**China** • 3♀♀; Jiangsu Province, Nanjing City, Jiangning District, Fangshan Scenic Area; 18 Jun. 2025; Xinyu Wang leg.; FAFU-1426, FAFU-1427, FAFU-1428.

**Figure 3.**
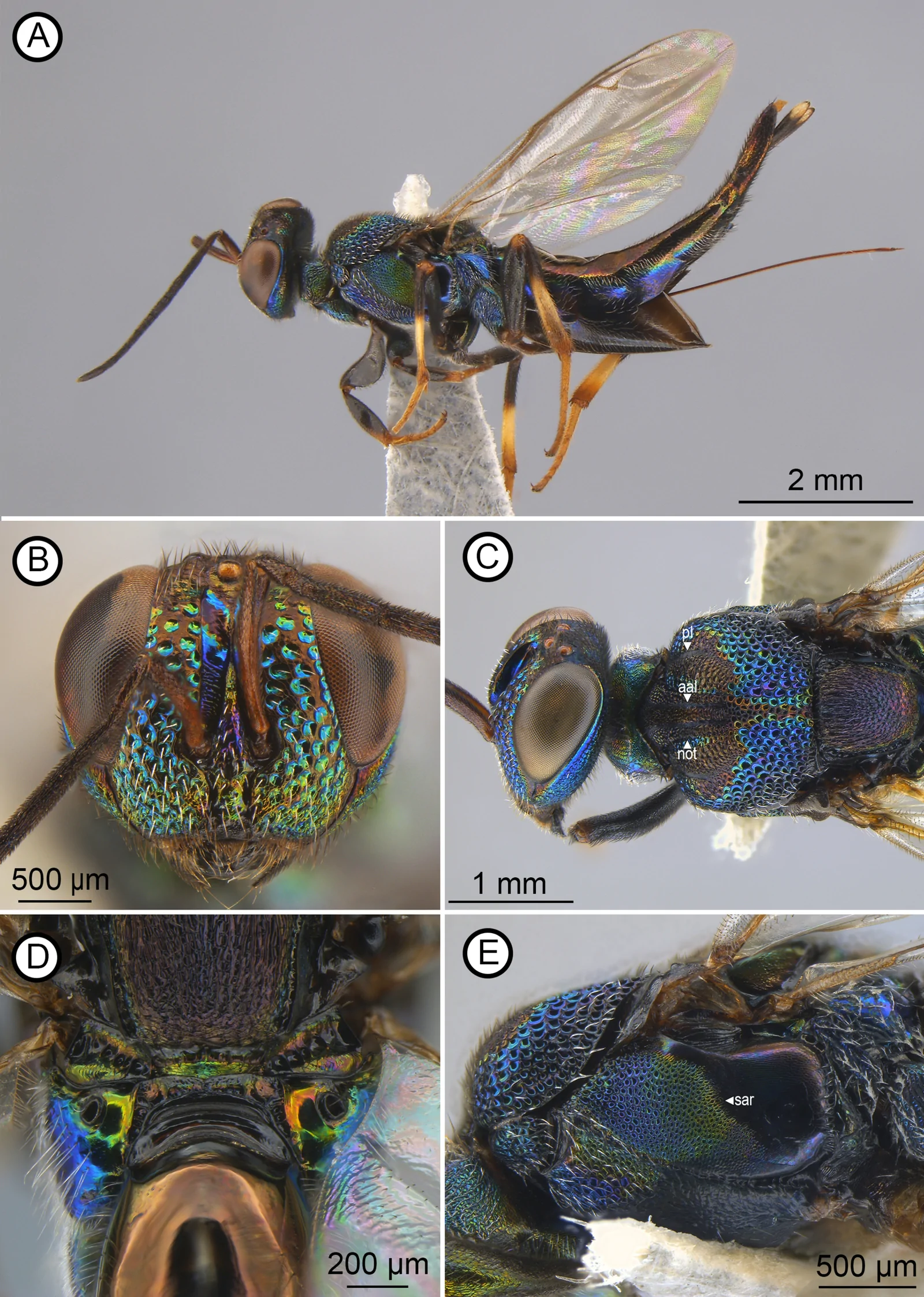
*Balcha indica* (Mani & Kaul, 1973), new record. **A**. Body, lateral; **B**. Head, frontal; **C**. Mesosoma, dorsal; **D**. Metanotum and propodeum; **E**. Mesosoma, lateral. (**A, B** from FAFU-1427; **C, E** from FAFU-1426; **D** from FAFU-1428). Abbreviations: aal = anteroadmedian line; not = notaulus; pl = parapsidal line; sar = subalar region.

##### Photograph.

**China** • 1♀; Jiangsu Province, Nanjing City, 18 June. 2025, photo by Xinyu Wang.

##### Diagnosis.

**Female**. Face (Fig. 3B) with punctures brightly colored in contrast to dark interstices, upper parascrobal region flat with shallow punctures, and uniformly setose. Mesoscutum (Fig. 3C) with dark coppery anteroadmedian and parapsidal bands forming Ψ-like pattern because of distinct bluish-green paranotaular bands; with slightly lanceolate white setae laterally, with dark hairlike setae dorsally, and with a few white lanceolate setae posteromedially. Scutellum (Fig. 3C) mostly dark or with slight coppery luster under some angles of light; reticulate posteriorly, and sculpture anteriorly aligned into longitudinal furrows and irregular ridgelike interstices. Metanotal panel (Fig. 3D) with single line of setae near anterior margin of precrenular region. Dorsellum (Fig. 3D) thick with posterior surface setose. Acropleuron (Fig. 3E) with postalar region alutaceous. Propodeum (Fig. 3D) with paraspiracular region bare. Fore wing (Fig. 3A) hyaline. Petiole (Fig. 3D) without longitudinal crenulation. Syntergum (Fig. 3A) shorter than length of mesosoma.

##### Distribution.

Canada (introduced), *China (Jiangsu), India, Myanmar, Thailand, USA (introduced), Vietnam.

##### Biology.

Two species, *Agrilus planipennis* Faimaire and *A. auroguttatus* Schaeffer (Coleoptera, Buprestidae) were recorded as hosts of *Balcha indica* in the USA (Gibson 2005; Tamm et al. 2025). In China, although its hosts remain unidentified, this parasitoid is associated with wood-boring insects infesting paper mulberry, *Broussonetia papyrifera* (L.) L’Hér. ex Vent.

##### Remarks.

The morphology of Chinese *Balcha indica* specimens examined in this study aligns with that of the Nearctic specimens described by Gibson (2005), but differs from other Old World material examined in his revision. Accordingly, the diagnosis of this species does not adequately encompass *B. indica* populations from all geographic regions; for a detailed morphological description, see Gibson (2005). Further relevant information is provided in the Discussion.

#### Balcha laciniosa

Taxon classificationAnimaliaHymenopteraEupelmidae

Gibson, 2005

7310E5FD-4963-5481-872D-0807A71394FF

Fig. 4

Balcha laciniosa Gibson, 2005: 36–39.

##### Material examined.

**China** • 1♀; Guangdong Province, Shaoguan City, Ruyuan Yao Autonomous County, Nanling National Forest Park alpine meadow; alt. 1545 m; 28 Sep. 2022; Wenfeng Li leg.; Malaise trap; FAFU-1208.

**Figure 4.**
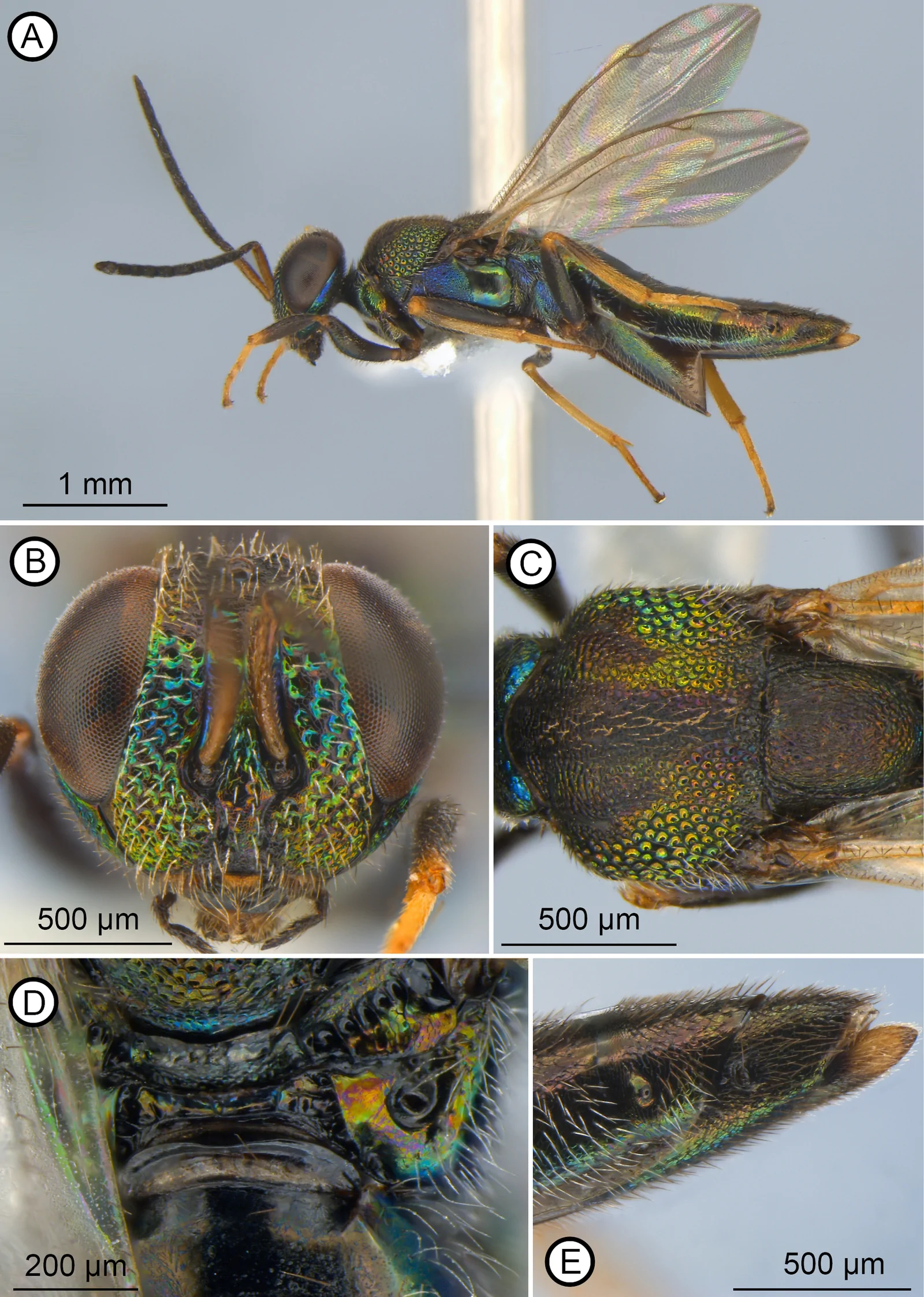
*Balcha laciniosa* Gibson, 2005, new record. (FAFU-1208). **A**. Body, lateral; **B**. Head, frontal; **C**. Mesosoma, dorsal; **D**. Metanotum and propodeum; **E**. Syntergum, lateral.

##### Diagnosis.

**Female**. Face (Fig. 4B) with punctures brightly colored in contrast to dark interstices. Mesoscutum (Fig. 4C) with anteroadmedian and parapsidal bands either forming distinct Ψ-like pattern but parapsidal band separated posteriorly from anteroadmedian band; with distinct, white, lanceolate setae laterally and dorsomedially within anteroadmedian band, the lanceolate setae dorsally interspersed with hairlike setae. Scutellum (Fig. 4C) mostly dark coppery with sculpture aligned into longitudinal rugae at least anteromedially, otherwise reticulate. Dorsellum (Fig. 4D) thick with posterior surface bare. Propodeum (Fig. 4D) with paraspiracular region bare. Fore wing (Fig. 4A) hyaline. Petiole (Fig. 4D) without longitudinal crenulation. Syntergum (Fig. 4E) consisting of undifferentiated tergum, surrounding all of the ovipositor sheath except the apex; uniformly sculptured and setose.

##### Distribution.

*China (Guangdong), Indonesia, Malaysia, Philippines, Vietnam.

##### Biology.

According to the original description (Gibson 2005), in Indonesia this species is a parasitoid of the larvae of *Agrilus kalshoveni* Obenberger (Coleoptera, Buprestidae).

##### Remarks.

The diagnosis of *Balcha laciniosa* is based on a typical, unaltered specimen characterized by naturally white dorsomedial mesoscutal setae. This character is not fully congruent with the specimen illustrated herein (Fig. 4C) due to staining during specimen preparation and preservation, resulting in a slight yellowish tinge of the setae. Additionally, the syntergum (Fig. 4E) of this individual exhibits intraspecific variation. Although *B. laciniosa* already possesses one of the shortest synterga within the genus, the syntergum of the examined specimen is even shorter than that reported in the original description, appearing nearly as long as high in lateral view.

#### Balcha reticulata

Taxon classificationAnimaliaHymenopteraEupelmidae

(Nikol’skaya, 1952)

7D587580-A226-532A-9D39-79B4C3664B50

Fig. 5

Calosota reticulata Nikol’skaya, 1952: 480.
Sauteria reticulata : Kalina 1984: 11.
Balcha reticulata : Gibson 2005: 46–48.

##### Material examined.

**China** • 2♀♀; Liaoning Province, Benxi City; 9 Jan. 2015; reared from *Larix gmelinii* (Rupr.) Kuzen.

**Figure 5.**
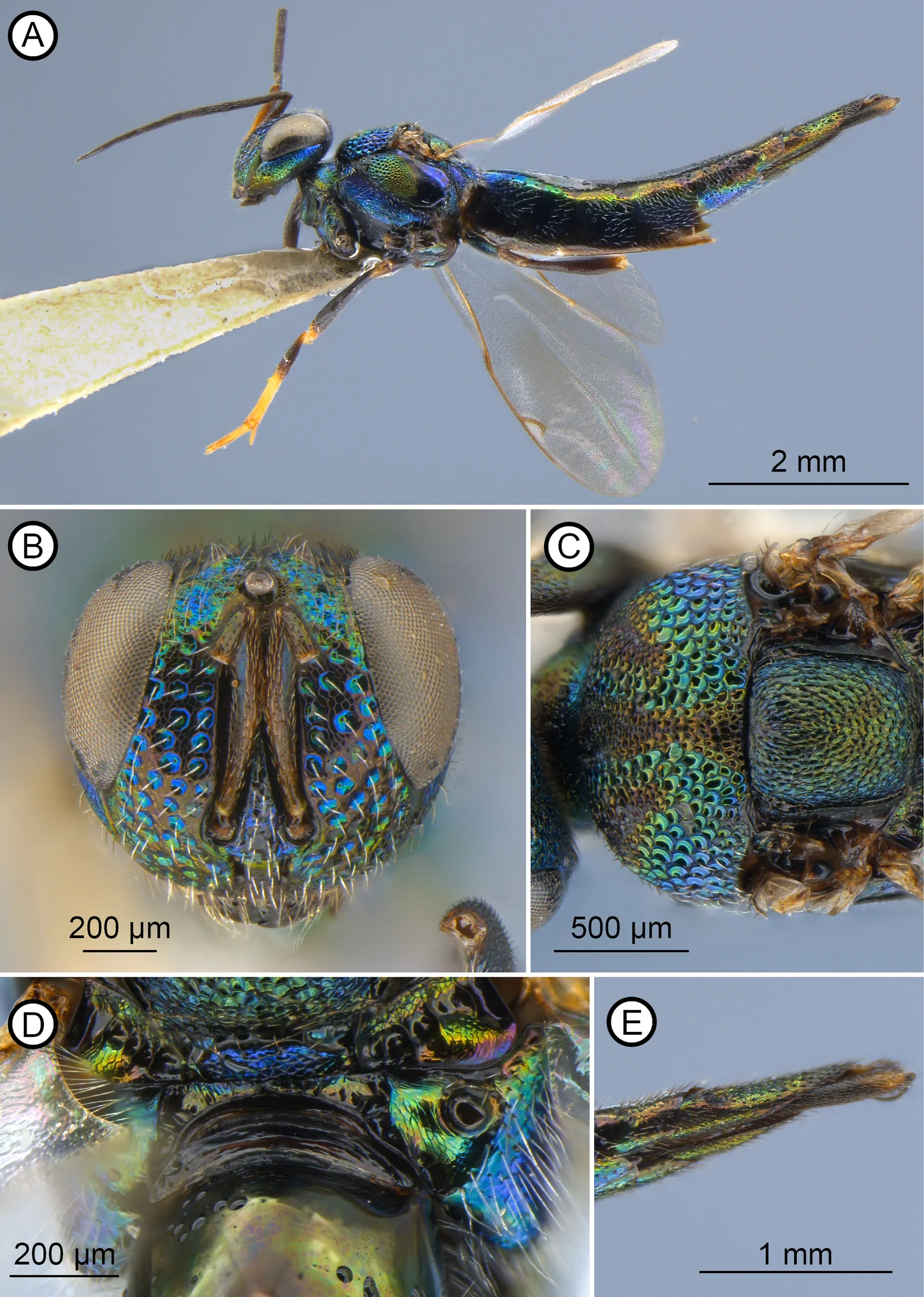
*Balcha reticulata* (Nikol’skaya, 1952), new record. **A**. Body, lateral; **B**. Head, frontal; **C**. Mesosoma, dorsal; **D**. Metanotum and propodeum; **E**. Syntergum, lateral. (**A, C, D, E** from BX-1; **B** from BX-2).

##### Diagnosis.

**Female**. Face (Fig. 5B) with punctures brightly colored in contrast to dark interstices. Mesoscutum (Fig. 5C) with three separate longitudinal coppery bands, the anteroadmedian band extending for length of mesoscutum and parapsidal bands extending ~1/2 length of mesoscutum; with brownish setae dorsally, and longer and slightly lanceolate white setae laterally. Scutellum (Fig. 5C) mostly greenish with variably distinct coppery luster, reticulate. Dorsellum (Fig. 5D) thick with posterior surface bare. Propodeum (Fig. 5D) with paraspiracular region bare, coriaceous. Fore wing (Fig. 5A) hyaline. Petiole (Fig. 5D) without longitudinal crenulation. Syntergum (Fig. 5E) consisting of undifferentiated tergum, surrounding all of the ovipositor sheath except the apex; uniformly sculptured and setose.

##### Distribution.

*China (Liaoning), Russia.

##### Biology.

Hosts unknown, but the examined specimens were reared from dead tree trunk of dahurian larch, *Larix gmelinii* (Rupr.) Kuzen.

##### Remarks.

*Balcha reticulata* resembles the holotype of *B. dictyota* in the color pattern of the mesoscutum and scutellum. However, the sculpture of the mesoscutum and scutellum is similar to that of both the holotype of *B. dictyota* and conspecific specimens from southern China. *Balcha reticulata* can be readily distinguished by its bare paraspiracular region (Fig. 5D), non-crenulate petiole (Fig. 5D), and face with brightly colored punctures on the parascrobal region that contrast with dark interstices (Fig. 5B).

## Discussion

### Origin and identity of *Balcha indica*

To infer the likely native range of North American *Balcha indica* populations, Gibson (2005) compared Nearctic and Old World specimens, focusing on three key morphological characters: leg coloration, mesoscutal setal patterns, and the sculpture of the mesoscutum, scutellum, and acropleuron. Two type specimens from northern India, together with a female specimen from Thailand, exhibit conspicuous divergence from Nearctic specimens. Furthermore, the Nearctic specimens diverge from other Old World specimens, exhibiting distinct differences in at least one morphological character. In this study, we compared Chinese *B. indica* specimens with Nearctic material. The Chinese specimens closely match the Nearctic specimens across all examined characters, sharing the following morphological features: dark femora (Fig. 3A), a mesoscutum that is dorsally much more uniformly alveolate with a few white lanceolate setae posteromedially (Fig. 3C, E), a scutellum with quite conspicuous anterior rugae (Fig. 3C), and an entirely alutaceous postalar region of the acropleuron (Fig. 3E). Furthermore, the COI gene sequence of *B. indica* generated in this study shows a pairwise K2P distance of only 0.2% with the COI gene sequences of *B. indica* specimens from North America, which were identified by Gibson and deposited in BOLD Systems. Notably, the host *Agrilus planipennis* and its ash tree hosts (*Fraxinus* spp.) are also naturally distributed in China. Collectively, these morphological, molecular, and host distribution lines of evidence indicate that the Nearctic population of *B. indica* most likely originated from China via commercial commodities, rather than from Vietnam or other parts of Southeast Asia via military transport.

### Sexual dimorphism and Intraspecific variation in *Balcha dictyota*

**Figure 6.**
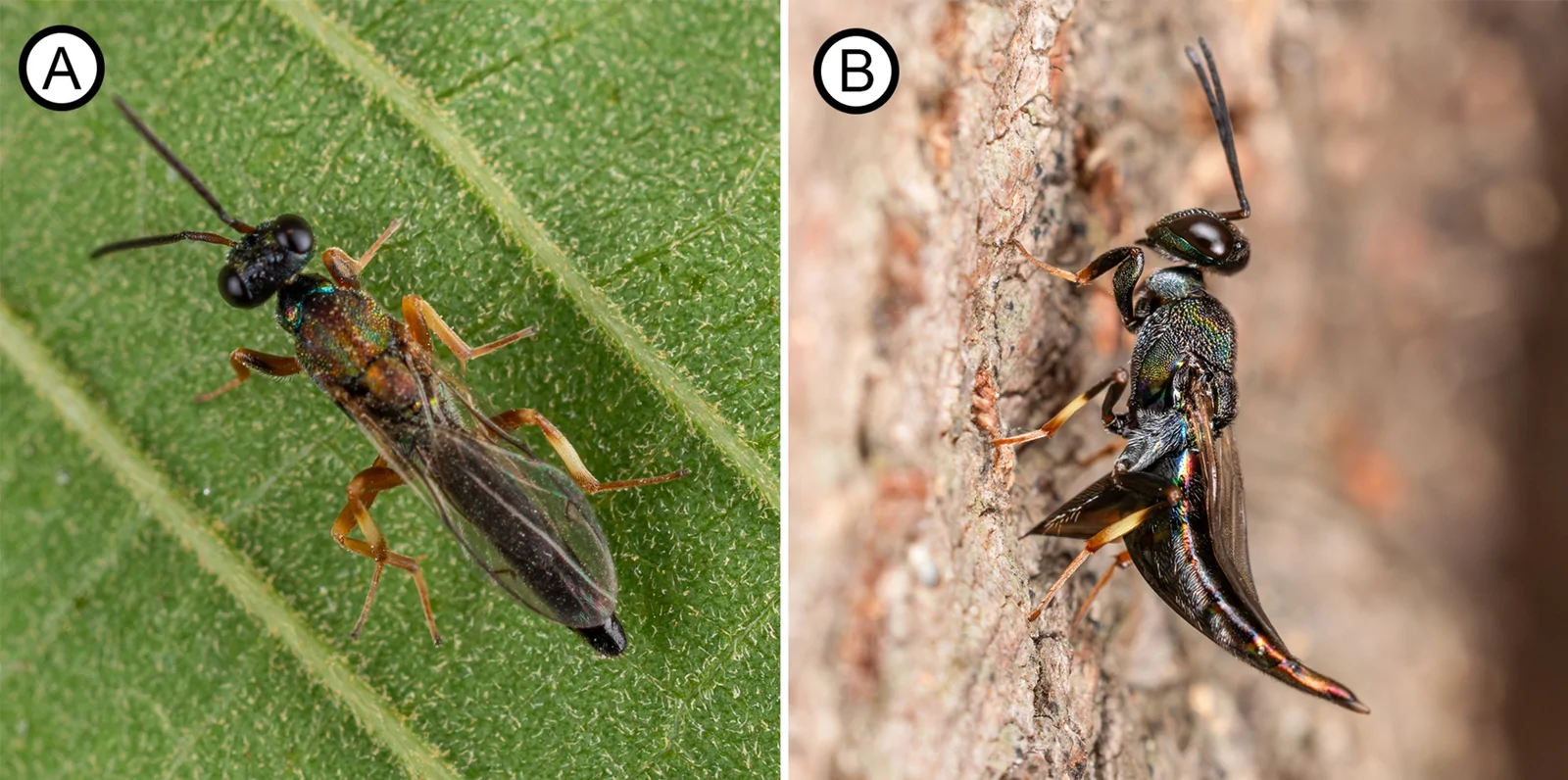
Field photographs by Xinyu Wang, Nanjing, Jiangsu, China. **A**. *Balcha dictyota*, male, 13 April 2025; **B**. *Balcha indica*, female, 18 June 2025.

Fusu et al. (2018a) supplemented the description of the *B. dictyota* male from South Korea and hypothesized slight sexual dimorphism in this species. Given that no males had been reported prior to their study, their speculation, based on a single male, was reasonable at the time. However, a *Balcha* individual recorded from Nanjing, Jiangsu Province (Fig. 6A) can be confidently identified as a male of *B. dictyota* based on its mesoscutum with conspicuously separated anteroadmedian and parapsidal bands, and scutellum and leg coloration, all consistent with females from southern China. This differs from the South Korean male (Fusu et al. 2018a: fig. 3E), in which the anteroadmedian and parapsidal bands appear connected mesally. Therefore, this morphological character state of the South Korean male can be excluded as sexual dimorphism and is instead attributed to intraspecific color variation. Unfortunately, the available photograph (Fig. 6A) is out of focus on the dorsum, and the specimen was not collected. Thus, no further discussion of the variation in the scutellum can be conducted for this species.

### Intraspecific genetic differentiation of *Balcha dictyota*

Beyond the intraspecific morphological variation documented above, our molecular data further revealed substantial intraspecific genetic differentiation within *B. dictyota*. The pairwise genetic distance of the COI gene region between the two successfully sequenced conspecific specimens reached 6.5%. Despite this high genetic divergence, both sequenced specimens were unambiguously assigned to *B. dictyota* through morphological examination, with all diagnostic characters fully consistent with the original species description (Gibson 2005), as well as the extensive intraspecific morphological variation documented both in Fusu et al. (2018a) and in the specimens examined herein (Fig. 1). Notably, such an elevated level of intraspecific COI gene divergence is not unprecedented within Eupelmidae. For instance, Tang et al. (2022) reported a 6.2% intraspecific COI gene distance in *Zaischnopsis lii* Jiang & Peng, 2022, a species with stable diagnostic morphological traits despite high mitochondrial divergence, confirming that pronounced intraspecific COI gene variation occurs in other eupelmid lineages. Even so, due to the extremely limited number of *B. dictyota* specimens available globally and the near absence of published molecular data for the genus as a whole, the full range of intraspecific genetic and morphological variation in *B. dictyota*, as well as the reliable intraspecific genetic distance threshold for species delimitation in *Balcha*, remain poorly resolved. Further studies incorporating more specimens across the entire distribution range of this species and additional molecular data for congeneric taxa are required to resolve these uncertainties.

### Host associations

Host relationships of *Balcha* are extremely poorly understood. Prior to this study, only three species had relevant biological records: specific host associations have been documented for *B. indica* and *B. laciniosa*, both parasitizing *Agrilus* spp. (see Biology subsection under the respective species in the Taxonomy section above); for the third species, *B. elegans*, only plant associations have been recorded, and its exact host remains unknown. This study documents plant associations for two additional *Balcha* species, supplementing existing biological information on *B. indica*, and fills a gap in our understanding of *B. reticulata*.

Three *B. indica* specimens were collected from trunks of *Broussonetia papyrifera*, a tree native to China. The collector observed the oviposition behavior in the field (Fig. 6B), and recorded various wood-boring beetles on these trees, including jewel beetles (Buprestidae), longhorn beetles (Cerambycidae), weevils (including bark beetles, Curculionidae); no wood-boring moths were observed. Based on the known biology of *B. indica*, its host is most likely jewel beetles, but parasitism of other wood-boring beetles cannot be excluded.

**Figure 7.**
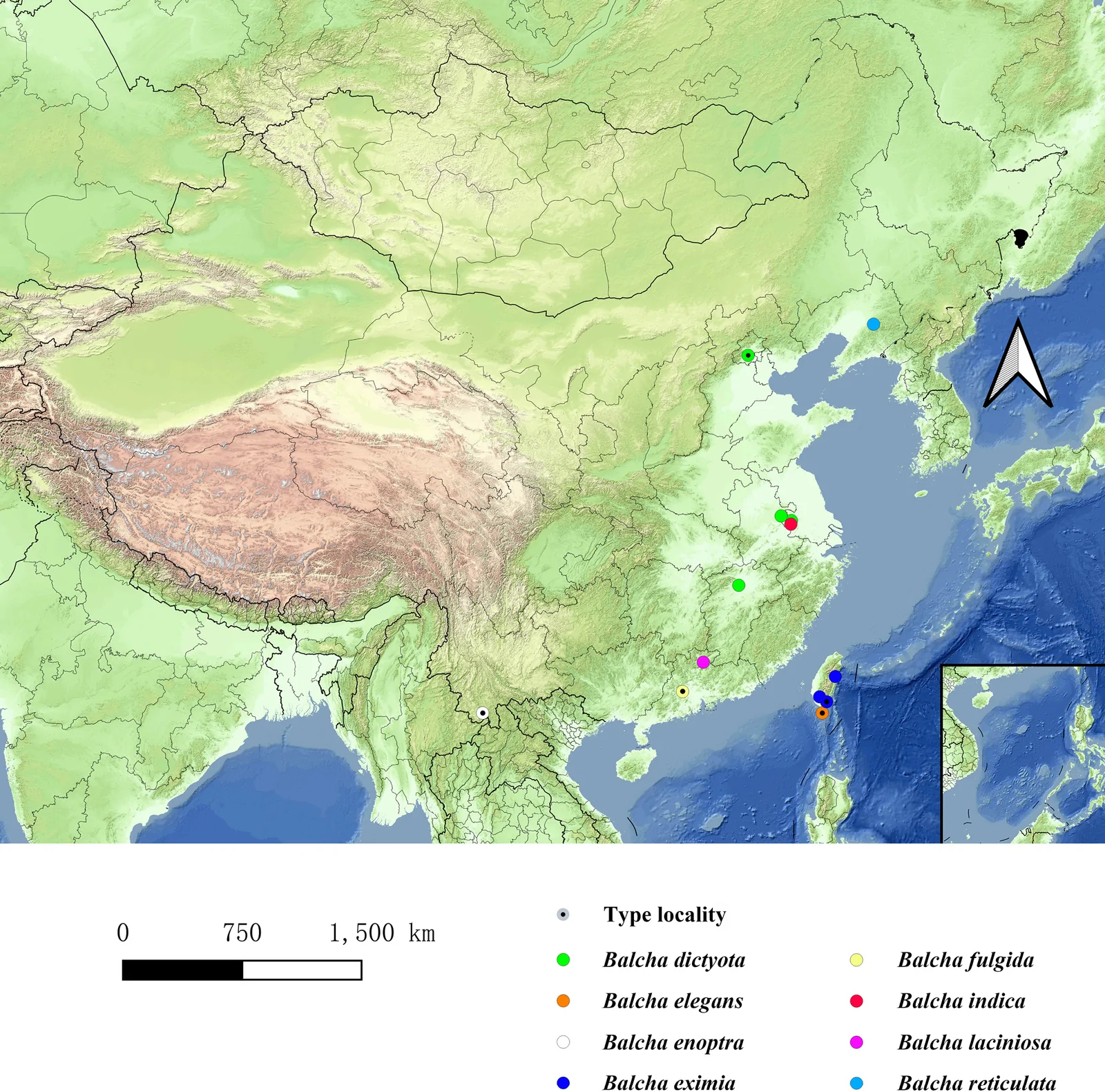
Distribution of Chinese *Balcha*.

Collection and emergence information for the two *B. reticulata* specimens was derived from their labels. The collector excised a section of *Larix gmelinii* trunk from Benxi, Liaoning Province (Fig. 7), transported it to the laboratory, and two *B. reticulata* individuals emerged from it on 9 January 2015. Based on the biology of *Balcha*, the host is speculated to be a wood-boring beetle associated with *L. gmelinii*. Since *L. gmelinii* is also distributed in the Russian Far East, it is reasonable to hypothesize that *B. reticulata* may also parasitize wood-boring beetles associated with this tree species in that region.

### Distribution

A distribution map of *Balcha* species in China (Fig. 7) was constructed based on our material and published records from Gibson (2005), which highlights the considerable potential for undiscovered species diversity within the country. Beyond this regional focus, a broader synthesis of the global distribution of the genus was compiled using citizen-science observations from iNaturalist and previously published records. Among these, records from Japan are consistent with the known East Asian distribution of the genus. In contrast, records from Poland and European Russia (Moscow) are highly unexpected: not only are these localities situated further north than the previously known northernmost distribution limit (at ca 43°N in the easternmost Russian Far East), but the genus has not been formally recorded from the European biogeographic realm previously. This study confirms China as a global diversity hotspot for *Balcha*, while the unexpected extralimital records underscore the need for a comprehensive re-examination of the global distribution and ecology of this genus.

## Supplementary Material

XML Treatment for Balcha

XML Treatment for Balcha dictyota

XML Treatment for Balcha fulgida

XML Treatment for Balcha indica

XML Treatment for Balcha laciniosa

XML Treatment for Balcha reticulata

## Additional information

### Artificial Intelligence (AI) use

The authors accept full responsibility for the content of the manuscript, including the disclosure of any use of AI.

No AI tools were used in the preparation of this manuscript.

### Author ORCIDs

Q. Wang https://orcid.org/0009-0005-3980-596X

H. Liao https://orcid.org/0009-0007-7856-8674

Z. Li https://orcid.org/0009-0009-2075-7020

Y. Zhang https://orcid.org/0009-0008-9216-1691

L. Peng https://orcid.org/0000-0002-3224-2675

### Data availability

All of the data that support the findings of this study are available in the main text.
